# Yawning reduces facial temperature in the high-yawning subline of Sprague-Dawley rats

**DOI:** 10.1186/s12868-016-0330-3

**Published:** 2017-01-03

**Authors:** Jose R. Eguibar, Carlos A. Uribe, Carmen Cortes, Amando Bautista, Andrew C. Gallup

**Affiliations:** 1 Research Office of the Vice Rectory of Research and Postgraduate Studies, Benemérita Universidad Autónoma de Puebla, 4 Sur # 104. Col. Centro, 72000 Puebla, Pue. Mexico; 2 Institute of Physiology, Benemérita Universidad Autónoma de Puebla, Apdo. Postal 5-66, Col. Prados Agua Azul, 72430 Puebla, Pue. Mexico; 3 Centro Tlaxcala de Biología de la Conducta, Universidad Autónoma de Tlaxcala, Tlaxcala, Mexico; 4 Psychology Department, SUNY College at Oneonta, Oneonta, NY USA

**Keywords:** Thermoregulation, Head cooling, Thermography, Brain circulation, Grooming, Anxiety

## Abstract

**Background:**

Yawning is a stereotyped behavior that enhances blood flow to the skull, and the resulting counterflow has been hypothesized as a mechanism for brain cooling. Studies have shown that yawns are strongly associated with physiological and pathological conditions that increase brain temperature, and that they are followed by equivalent decreases in brain temperature. However, measured reductions in cranial or facial temperatures following yawning have yet to be reported, to our knowledge. To accomplish this, we used a subline of Sprague-Dawley rats that yawn at a much greater rate (20 yawns/h) than do outbred Sprague-Dawley rats (2 yawns/h).

**Results:**

Using an infrared camera, we effectively evaluated thermal changes in the cornea and concha of these rats before, during, and after yawns. The maximum temperature in both regions significantly decreased 10 s following yawns (concha: −0.3 °C, cornea: −0.4 °C), with a return to basal temperatures after 20 s.

**Conclusions:**

This study is the first clear demonstration of yawning-induced thermal cooling on the surface of the face, providing convergent evidence that this behavior plays a functional role in thermoregulation. As other studies have demonstrated that yawning is capable of reducing cortical brain temperature, our current data support the idea that yawning functions as a thermoregulator, affecting all structures within the head.

## Background

Yawning is an innate behavior characterized by a stereotyped motor pattern. Wide opening of the mouth and wide dilation of the pharynx and larynx is followed by deep inspiration that ends abruptly with a short expiration. Neck musculature then shrinks and returns to basal levels [1, 2]. Understanding the physiological significance of yawning is important because spontaneous yawning is ubiquitous in vertebrates [3], with reports identifying atypical yawning as a symptom or side-effect of several neurological diseases or medications [4]. Many hypotheses have been proposed regarding the potential physiological functions of yawning; however, they often lack clear physiological evidence [5]. While much of the scientific community believed that yawns serve a respiratory function, experimental procedures performed on humans have demonstrated that yawning and breathing are controlled by separate mechanisms [6]. However, other causes such as fatigue, boredom, and sleepiness have been proposed as causes of increased yawning frequency [4]. One recent theory that has gained recent experimental support proposes that yawning is a brain cooling mechanism [7–9].

In homeotherms, brain temperature is determined by three variables: the rate of arterial blood flow, the temperature of arterial blood, and the amount of metabolic heat production [10]. The physical action of yawning can alter the first two variables. Yawning produces significant changes in circulation, including accelerating heart rate by up to 10 additional beats per minute [11, 12]. The deep inhalation and powerful jaw stretching during a yawn also favors increases in blood flow to the skull [13], and the extended contraction of the lateral pterygoid muscle during yawning acts to squeeze blood from the associated plexus [14]. Therefore, the gaping of the jaw and deep inhalation increase arterial blood flow to the brain and enhance venous return, thus acting to remove hyperthermic blood from the skull and simultaneously introduce cooler blood coming from the lungs and extremities. Increased facial blood circulation and associated changes in the ventilation rate are two well-known mechanisms that cool brain temperature [15, 16].

The deep inhalation of air that accompanies yawning may also provide counter-current heat exchange by cooling venous blood draining from the nasal and oral orifices that is in close contact with arterial blood supply [16]. Consistent with this view, recent studies in human and non-human primates [8, 17] have shown that pharyngeal cooling rapidly and selectively decreases brain and tympanic temperature by cooling the carotid arteries [17]. Furthermore, anatomical investigations in humans have revealed that the thin sinus walls flex when pterygoid musculature contracts during yawning, indicating that the posterior wall of the maxillary sinus serves as an origin for both medial and lateral pterygoid muscle segments [18]. This powerful flexing of the sinus walls has been proposed to ventilate the human sinus system similarly to what occurs in birds [19], providing a second mechanism through which yawning functions in human cerebral cooling. Accordingly, yawning could reduce brain temperature by ventilating the sinus system and promoting the evaporation of the sinus mucosa [20]. The salivation and tearing that accompany yawning could be a third mechanism for cooling, aiding in heat loss through evaporation [8].

Importantly, yawning appears to be constrained to a narrow range of ambient temperature (i.e., a thermal window). Numerous reports have confirmed and replicated the specific thermal window for various species. For heat to dissipate; ambient temperatures must be lower than the body temperature of the subject animal. Thus, as predicted, in warmer environmental situations, yawning did not cool the brain [7–9, 20]. Consistent with models of thermoregulation, variation in ambient temperature elicits predicted fluctuations in yawning frequency in a number of species, including budgerigars [21], rats [22], white-faced capuchin monkeys [23], and humans [20, 24].

Initial rises in temperature within this window trigger yawning and other thermoregulatory cooling mechanisms such as panting and behavioral adjustments to reach thermoneutralization. However, as external temperatures continue to rise beyond the thermal window, yawning decreases because counter-current heat exchange becomes less effective. Similarly, yawning is also reduced at extremely cold temperatures, as thermoregulatory cooling responses are no longer necessary [24].

Direct measures of internal temperature in rats and humans have shown that brain and oral temperature rise immediately before the onset of yawns, with corresponding decreases in temperature being observed directly following these events [9, 25]. One interpretation is that yawns are triggered by elevated temperature inside the skull, and that the physiological consequences of yawning discussed above result in temperature reduction. Consistent with this view, heightened core body temperature following handling-stress has been shown to be correlated with earlier onset and higher frequency of yawning in birds [26], and experimental manipulations designed to promote brain cooling have been shown to diminish yawning frequency in humans [7]. Furthermore, a number of medical conditions and drugs affect yawning and brain/core temperature in reciprocal patterns. Conditions associated with rises in temperature present with increased yawning, while those associated with lower temperatures present with diminished yawning [8]. However, whether yawning reduces the temperature on the surface of the head or in the face remains unclear.

Selective breeding of Sprague-Dawley rats has generated a subline with a higher incidence of spontaneous yawning [27], which is a useful tool for the physiological study of yawning. Yawning frequency (20 yawns/h) in these high-yawning (HY) rats is an order of magnitude higher than in other Sprague-Dawley rats (2 yawns/h) [27–29]. Thus, HY animals allow us an efficient means to evaluate internal and environmental variables that modulate yawning frequency. Indeed, HY rats were used to determine that yawning has a circadian oscillation pattern that includes significantly increased yawning frequency when transitioning from light periods to dark periods [30]. Under constant light conditions, the circadian rhythm of yawning is disorganized, suggesting that this behavior is not endogenously generated [31]. However, restricting food access to just 2 h per day produced a highly significant increase in yawning frequency. This is because food access can be a predictor of food availability and can produce an anticipatory yawning peak [31].

High-yawning rats allow us to analyze the role of smell on contagious yawning [32]. HY rats are also more sensitive to cholinergic and dopaminergic agents that increase yawning frequency [33, 34], and to adrenocorticotrophic hormones and oxytocin neuropeptides [29, 35], indicating a higher sensitivity of these sublines to environmentally and pharmacologically induced yawning.

Based on past findings regarding yawning and thermoregulation, the present study used a thermographic imaging camera to measure temperature changes associated with spontaneous yawning that occurred in the cornea and concha of HY rats. The concha is a good candidate structure because the ears dissipate more heat than other head regions and act as a thermal buffer mechanism in many mammalian species. Similar structures such as the cornea play critical roles in thermoregulation, and are frequently called thermal dissipaters because they are responsible for heat exchange and they regulate surface temperature [36, 37].

During yawning, the upper airways are ventilated, and this possibly cools the blood in the facial artery, which travels around the mouth and nasal cavities, making terminations around the eye and cooling these structures [7]. Thus, the two anatomical areas that we selected are good candidates for directly detecting temperature change in areas not covered by fur.

## Results

### Spontaneous yawning frequency

**Fig. 1:**
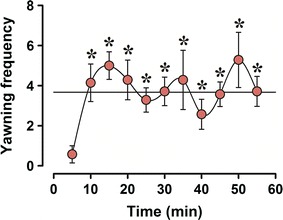
Higher expression of spontaneous yawning frequency in high-yawning (HY) subline is stable after adaptation period. HY rats yawned 283 times during the observation period (50 min). Yawning frequency was stable between 15 and 55 min, with a mean value of 3.7 yawns/5 min, being different only in the first 5-min period with respect to the whole observation period (*P < 0.05)

**Fig. 2:**
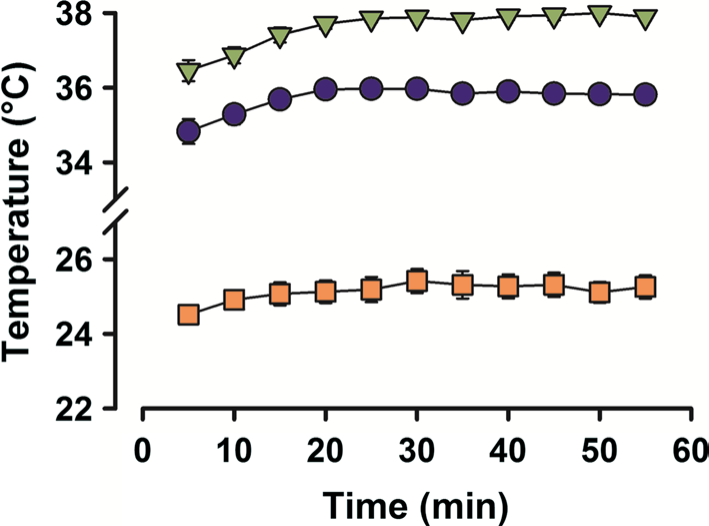
Temperatures in facial areas and in the observation box. Temperatures in the cornea (*blue circles*), concha (*green inverted triangles*), and inside the wooden box (*orange squares*) were stable between 15 and 55 min

Yawning frequency was calculated for each 5-min epoch of the 60-min observation period. The first 5-min epoch had a mean of 0.6 yawns, which increased and stabilized to 3.7 yawns/5-min epoch during the remaining 50 min of the observation period (Fig. 1). Yawning frequency in the first epoch was significantly different from all others (repeated ANOVA F_10_ = 2.3, *P* < 0.03, followed by Tukey’s test, *P* < 0.05). Importantly during the rise of ambient temperature, yawning frequency also increased, reinforcing the role of this behavior as a brain cooling mechanism. In contrast, yawning frequency was relatively constant during the subsequent intervals when the ambient temperature remained relatively unchanged. This is a prerequisite for evaluating changes in facial temperature closely associated with yawning in time. Because of this, temperature recordings were only evaluated for the period between 10 and 55 min (Fig. 2, orange squares).

### Temperature changes in the facial structures

We recorded 2667 thermographic frames and calculated mean temperature values for the concha and cornea for each 5-min epoch of the observation period. The mean corneal temperature was 34.8 ± 0.3 °C in the first epoch, rising 0.1 °C during the next 15 min to reach a maximum of 35.8 ± 0.2 °C, which remained stable for the remainder of the observation period (Fig. 2, blue circles). Corneal temperature during the first epoch was significantly different from the remaining epochs (ANOVA: F_10_ = 6.9, *P* < 0.001, followed by Tukey’s test *P* < 0.002, with respect to the first period). The mean conchal temperature was 36.5 °C in the first epoch, rising to a maximum of 37.9 ± 0.1 °C after 20 min (Fig. 2, green inverted triangles). Conchal temperatures during the first and second epochs were significantly different from those during the fourth through eleventh intervals (ANOVA: F_10_ = 15.5, *P* < 0.001, followed by Tukey’s test, *P* < 0.001).

To have control of variables, we simultaneously measured temperature values inside the wooden observation box at 5-min intervals to evaluate surrounding ambient temperature. The temperature inside the observation box was 24.5 ± 0.3 °C during the first epoch and reached a maximum of 25.4 ± 0.3 °C after 30 min (Fig. 2, orange squares). The first epoch was significantly different from the remaining epochs (ANOVA: F_10_ = 4.3, *P* < 0.001, followed by Tukey’s test, *P* < 0.03). Therefore, as with yawning frequency, temperatures for both facial structures and the surrounding environment increased steeply during the first interval and then became stable for the remaining time periods. Therefore, these intervals were selected for the thermographic analysis associated with yawning.

### Correlation between facial temperatures

**Fig. 3:**
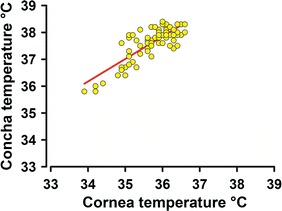
Correlation between corneal and conchal temperatures during the observation period. During the 45-min observation period, the facial temperatures at the two separate locations were significantly correlated (r = 0.81, *P* < 0.001)

To assess the consistency of temperature changes at the two measurement sites, we ran correlation analyses between the temperature changes in the cornea and concha across the testing sessions. Additionally, we compared the average temperatures between the left and right cornea. We found a very strong correlation between cornea and conchal temperatures (r = 0.81, *P* < 0.001; Fig. 3), and mean temperatures in the right and left cornea were virtually identical (35.5 ± 0.1 °C vs. 35.1 ± 0.1 °C; Student’s *t* test: t = 0.2, *P* = 0.8).

### Changes in corneal and conchal temperatures surrounding yawns

**Fig. 4:**
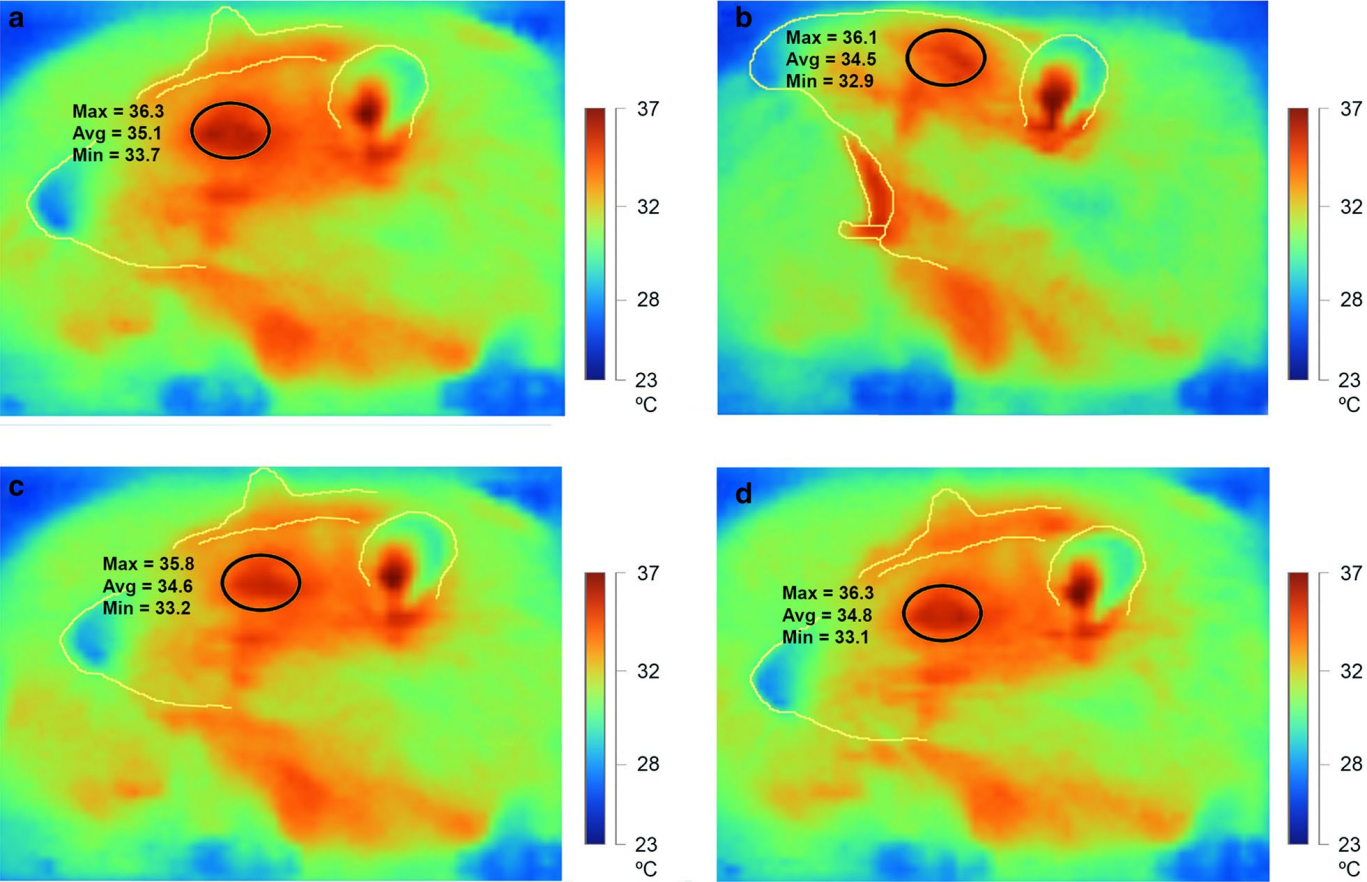
Representative corneal temperature changes during yawning using a thermographic camera. Serial thermographic pictures before, during and after a yawn. *Black circles* surrounding the cornea demonstrate the areas in which all measurements were taken. **a** 10 s before the onset of the yawn, the maximum (Max) temperature in the cornea was 36.3 °C. **b** When yawning, the Max temperature in the cornea dropped to 36.1 °C. **c** After 10 s, the temperature dropped even further, reaching the lowest Max temperature of 35.8 °C. **d** After an additional 20 s. Max temperature returned to the basal level at 36.3 °C. Temperature scale bars are on the *right side* of each *panel*

Two hundred and eighty-three yawns were recorded during the testing sessions. The individual yawn counts for the seven rats were as follows 40.4 ± 4.4 (mean ± SE). We only analyzed frames in which thermal evaluations were stable across rats and were taken from the same angle 10°, making it possible to compare changes in corneal temperatures (surrounded by black circles) before and after a yawn, as illustrated in thermographic images in Fig. 4.

**Fig. 5:**
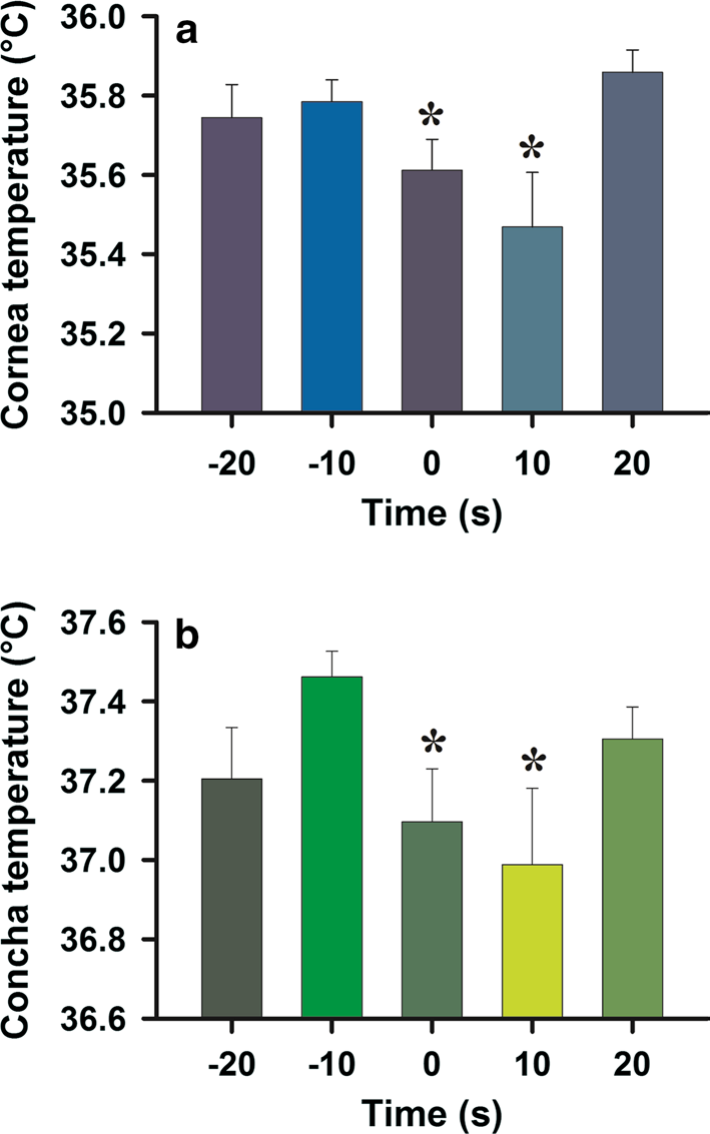
Average corneal temperature significantly changed around the time of yawning events. **a** Corneal temperatures were significantly different across the five time points (*P < 0.01). Temperatures significantly decreased during yawning and continued to decrease for 10 s. By 20 s after yawning, temperatures had begun to rise again. **b** Conchal temperatures were significantly different across the five time points (*P < 0.01). Temperatures increased before yawning, supporting the expression of yawning, and significantly decreased during yawning and the following 10 s. Subsequently they rose back to basal levels. All data points are the mean ± SE of at least 15 thermographic frames

To homogenize the data, temperatures greater than two standard deviations from the mean (<5%) were excluded from the analysis. We compared the data from five time intervals: 20 and 10 s before the yawn (labeled as −20 and −10, respectively, Fig. 5), during the yawn (0), 10 s following a yawn (10), and finally an interval 20 s following a yawn (20). The total number of frames evaluated in the five periods surrounding yawns were 55, 68, 56, 31, and 66.

Predicted changes in facial temperatures occurred surrounding yawning events. All temperatures measured (minimum, mean and maximum) were statistically different across the five time windows at both anatomical sites (cornea ANOVA: F_4_ = 4.0, *P* < 0.01; concha ANOVA F_4_ = 3.6, *P* < 0.01). Maximum corneal temperatures increased slightly from 35.74 ± 0.08 to 35.77 ± 0.05 °C (mean ± SE) between 20 and 10 s preceding yawns. Then, temperatures significantly decreased during yawns to 35.61 ± 0.07 °C (Tukey’s test, *P* < 0.05), reaching the lowest value of 35.46 ± 0.13 °C (Tukey’s test, *P* < 0.05) 10 s after the yawn. Subsequently, temperatures rose to 35.85 ± 0.05 °C by 20 s after yawns were completed (Fig. 5a). Similarly, maximum conchal temperature rose from 37.20 ± 0.12 to 37.45 ± 0.06 °C between 20 and 10 s preceding yawns. Temperatures significantly decreased during yawns to 37.09 ± 0.13 °C (Tukey’s test, *P* < 0.05: Fig. 5b), reaching the lowest temperature of 36.90 ± 0.19 °C (Tukey’s test, *P* < 0.05) 10 s after yawns. Subsequently, by 20 s after yawns were completed, temperature had increased to 37.3 ± 0.08 °C.

## Discussion

The use of infrared thermal imaging provides an effective means of tracking behavioral thermoregulation through changes in surface temperature [36, 37]. This is the first study to document fluctuations in facial temperature that are temporally locked to yawning, revealing that temperatures in the cornea and concha consistently rise leading up to yawning events as shown in Fig. 5b (−10 s) and then decrease below baseline levels shortly thereafter (10 s). These results are consistent with previous research measuring changes in brain temperature surrounding yawns in rats [25], and provide further evidence supporting the idea that yawning functions to cool the head and cortical surface [7–9].

Previous reports indicate that yawns are triggered by rises in brain and body temperature [21, 25, 38], which is consistent with the pattern of temperature change we observed preceding yawns. This is illustrated in Fig. 5b, in which 10 s before a yawn occurs, there is a small increase in the concha temperature that drops significantly during and 10 s after yawning. This is likely because this area is more exposed to thermal influences, as has already been demonstrated in other species [36, 37]. Because rat brain temperature is consistently higher than that of the arterial blood supplying the brain [39], increases in arterial blood flow and enhanced venous return that result from the act of yawning (i.e., wide opening of the mouth and deep inhalation) could explain the cooling we observed within 10 s of yawning. Heat convection by blood depends on several variables, including the volume and velocity of blood flow in the vessels [40]. For example, increased blood-flow velocity can result in a twofold increase in heat transmission by tissue [41], and specific reductions in brain temperature have been observed because of increased localized blood flow [42]. Therefore, the decreased temperatures that we observed at the corneal and conchal surfaces are consistent with various models of bio-heat transfer that take perfusion rates into account [43–46].

We must emphasize that the main heat regulator in rats and mice is the tail. However, facial surface area is also a well-known heat dissipater [47, 48]. Indeed, in humans, the eyeball—and in particular the cornea, iris, and sclera—are structures with high temperature conductivity because they often have higher temperatures than the environment [49]. Therefore, these eye structures function as thermal dissipaters that work through heat convection, and even evaporation, because tears allow the transmission of heat from inside the cranium to the surface of the eye [49].

The decreases in surface temperature at the cornea and concha sites correspond with previously documented changes in brain-tissue temperature following yawning events in rats. Shoup-Knox et al. [25] showed that temperature in the prelimbic cortex rise sharply just before yawns and then begin to drop after about 18 s, with maximum cooling occurring at about 60 s following this transition. This time course is much longer than what we observed externally on the eye and the ear, and metabolic heat production from neuronal activity inside the skull should interact with other structures and blood flow that can be responsible, at least in part, for the delayed responses. Together, these observations provide a consistent timeline for a functional role of yawning in cooling the brain, with temperature reductions at external surfaces preceding those at internal brain tissue. It is valid, therefore, to mention that both phenomena share common mechanisms and are temporally linked. Additional experiments are necessary to establish a causal relationship.

Cooling of external surfaces in the head might also contribute to cooling of internal tissues. In humans, the ophthalmic vein, like other emissary veins, has been reported to play an important role in cerebral cooling and may even reverse blood flow depending on the temperature conditions of the environment. Therefore, during hypothermia, blood travels from the brain to the surface of the face, whereas during hyperthermia the opposite pattern occurs [50]. Because yawning in rats and humans has already been demonstrated to be triggered by increases in brain and oral temperature [25, 38], cooling of external surfaces such as the eye and ear could directly alter brain temperature. Our observations of a mean reduction of −0.32 °C in the cornea and −0.48 °C in the concha are consistent with the reduction of just over −0.11 °C in the cerebral cortex of rats following yawns observed in a previous study [25]. The differences can be explained by the heat gain/loss ratio that follows circulation in blood vessels, as well as by differences in the quantity of blood supply to the brain and face, which are governed by different vascular tones and by evaporation in the paranasal sinuses [51]. Importantly, the corneal surface immediately returned to pre-yawning temperatures because blood in the ophthalmic artery carries heat continuously from inside the cranium, and in the case of the concha, heat-loss primarily results from convection and conduction mechanisms.

Importantly, Sato-Suzuki et al. [52] showed that chemical or electrical stimulation of the paraventricular nucleus of the hypothalamus evoked yawning and shifted EEG activity from delta to theta rhythms. They also showed an association between a drop in blood pressure and skin conductance supporting changes in the sympathetic inhibitory responses. Our results showed changes in the temperature of facial structures due to changes in the blood flow to the head. These changes are closely associated with the loss of heat in the oropharynx due to air flow, and support the validity of the thermoregulatory role of yawning.

## Conclusion

Our results are consistent with a growing number of comparative studies supporting the thermoregulatory theory of yawning [9]. The use of HY rats in this study provides an effective sample population for this investigation because of their high frequency of spontaneous yawning. However, further studies using similar thermal imaging techniques are needed to replicate these findings in other lines or species with more typical yawning patterns.

## Methods

### Animals

We used seven male HY Sprague-Dawley rats (5 months old, 470 ± 7 g) based on the premise that yawning is a sexually dimorphic pattern (spontaneous yawning rates have been shown to be 20 yawns/h for males, and less than 2 yawns/h for females) [53]. Rats were obtained from the animal room facilities at the Institute of Physiology at Benemérita Universidad Autónoma de Puebla, México, and were maintained under standard conditions with temperature 22 ± 1 °C and 30–45% relative humidity. Animals were housed 2–3 rats/acrylic cage, with free access to balanced rodent pellets (LabDiet, Rodent Diet 5012, USA) and purified water. The light–dark cycle was set to 12:12, with lights on at 0700. All rats were tested between the hours of 1100 and 1300 to avoid circadian changes [30]. All experiments were performed following the NIH Guide for the Care and Use of Laboratory Animals (NIH publications No. 80-23). This research was approved by the local Institutional Animal Care and Use Committee at Benemérita Universidad Autónoma de Puebla.

### Experimental design

We used an infrared camera (Fluke TiR1) for measuring surface temperature in the concha and in the cornea. The camera has a measurement range from −20 to +150 °C, with a thermographic resolution of 640 × 480 pixels. The camera uses SmartView™ software (Fluke Co., England) for detail assessment of temperatures and is capable of determining the maximum, minimum, and mean temperatures of a given area in a single image. Red zones in the concha and cornea were delineated with a 45-mm^2^ ellipse, considering that the surrounding areas are cooler because of the presence of skin covered by fur, as shown for the cornea in Fig. 4, with a black circle surrounding the cornea measured over red areas with higher temperatures and the same pattern with the concha. We used the measurements for the minimum, mean and maximum temperatures because the camera provided high sensitivity and resolution with an accuracy of 0.1 °C. This area was consistent, and results equivalent, when a blind observer performed the procedure. Temperature recordings were included in the analysis only when the images showed an angle of less than 10° of variation around the position of the nose. We also measured temperatures at the bottom of the observation box to control ambient temperature conditions throughout the experiment because this is an important variable for heat exchange mechanisms.

### Procedure

Rats were transferred to a soundproof room with controlled temperature and humidity, illuminated by two overhead incandescent lamps (220 lx). They were adapted to the observation conditions by living 1 h for 3 consecutive days in a wooden box (21 × 11 × 7 cm) that partially restrains their movements and allows a clear observation of yawning. This adaptation process is necessary to reduce stress responses on the test day. On the 4th day, we evaluated yawning frequency simultaneously with changes in temperature at the cornea and concha. Under these circumstances, we could take serial thermographs at 10-s intervals. A researcher situated in front of the wooden observation box aligned the infrared camera to line up exactly in front of each rat at a constant distance of 30 cm, allowing us to delineate and measure the same area around the concha and cornea for all measurements. This is an important control to maintain conditions with respect to the heat sources and reflections, including the wooden box itself. Because infrared is invisible to humans, we always recorded a pair of measurements before and after the experimental sessions to measure temperatures under control conditions.

After a 5-min adaptation period, we evaluated spontaneous yawning frequency for 55 min and manually took thermographic images at roughly 10-s intervals independently of the behavior for each animal. A second blind observer recorded the time when the image was taken and when yawns occurred using Observer XT software (Noldus, Netherlands).

### Statistics

Offline analyses allowed us to pair each yawning event with changes in the -conchal and corneal temperatures. All data were evaluated using Sigma-Plot v. 12.0 (Systat software, USA), and the statistical analyses were performed using the statistics module of Sigma-Plot. Comparison tests included a repeated analysis of variance (ANOVA) followed by Tukey’s test with *P* < 0.05 considered statistically significant.

## Abbreviations

HY: high-yawning
ANOVA: analysis of variance
NIH: National Institutes of Health
